# Evaluation of Pulmonary Function in Post-convalescent COVID-19 Adults: A Comparative Insight

**DOI:** 10.7759/cureus.61773

**Published:** 2024-06-06

**Authors:** Ghada E Elgarawany, Sapna Shevade, Shahad Aldebi, Bader Ahmed, Fagr Omer, Raghad Adel

**Affiliations:** 1 Department of Biomedical Science, College of Medicine, Gulf Medical University, Ajman, ARE; 2 Department of Medical Physiology, Faculty of Medicine, Menoufia University, Shibin El Kom, EGY

**Keywords:** peak expiratory flow rate, forced vital capacity, forced expiratory volume 1, spirometry, covid-19 infection, pulmonary function test

## Abstract

Background and objective

COVID-19 is a respiratory disease that is highly contagious and is caused by severe acute respiratory syndrome coronavirus 2 (SARS-CoV-2). Symptoms vary from mild to severe, where most of the patients suffer from high fever, severe headaches, dry cough, and exhaustion, while the less common symptoms are diarrhea, loss of taste, sore throat, and loss of smell. Following recovery from COVID-19, some patients displayed a restricted pattern in the function of their lungs. As a result, documenting the effects of COVID-19 after infection is essential since it provides a better understanding of the long-term consequences of COVID-19. Hence, the objective of the present study was to assess pulmonary functions in post-convalescent COVID-19 patients.

Methodology

A cross-sectional comparative study was conducted among students and staff members of Gulf Medical University for a duration of one year from 2021 to 2022. Through a convenient sampling method, a total of 100 participants were recruited for the present study, in which pulmonary function tests (PFTs) were performed using a spirometer, and O2 levels were measured using a pulse oximeter. Additionally, respiratory rate and pulse rate were monitored.

Results

The present study highlighted the comparison of PFTs in post-convalescent COVID-19 patients and concluded that smoker and convalescent COVID-19 groups showed non-significant decrease (p>0.05) in forced vital capacity (FVC) prediction, forced expiratory volume in the first second (FEV1) prediction, FEV1/FVC%, forced mid-expiratory flow rate (FEF_25-75%_) prediction, peak expiratory flow rate (PEFR) prediction, respiratory rate, and pulse rate in comparison to the control group. In comparison to the convalescent COVID-19 group, convalescent COVID-19 smoking patients showed a significant increase in FEV1/FVC% (p=0.04). Additionally, in comparison to the convalescent COVID-19 group, a significant increase in PEFR prediction values was observed with a p-value of 0.045 and in comparison to the smoker group with a p-value of 0.006. Moreover, oxygen saturation (SpO2) levels demonstrated non-significant changes between the groups.

Conclusion

The study concluded that for FEV1/FVC% and PEFR prediction values among the convalescent COVID-19 smoking patient group, a significant increase was observed in comparison to the convalescent COVID-19 group. This aids healthcare professionals in amending strategies to prevent consequences resulting from post-COVID-19 infection.

## Introduction

COVID-19 is an infectious disease caused by coronavirus [1,2], and severe acute respiratory syndrome coronavirus 2 (SARS-CoV-2) leads to this extremely contagious respiratory disease. This virus is believed to have originated from animals and spread to humans through person-to-person contact, leading to the most recent pandemic [3,4]. Similar to other viruses, COVID-19 symptoms vary from mild to severe, where most of the patients suffer from high fever, severe headaches, dry cough, and exhaustion, while the less common symptoms are loss of smell, diarrhea, sore throat, and loss of taste. In severe cases, COVID-19 may lead to inflammation of the alveoli and eventually may cause pneumonia. As pneumonia progresses, patients may suffer from shortness of breath and acute respiratory distress syndrome [4,5].

COVID-19 has been a prominent part of the past three years, claiming the lives of millions of people. Many patients with post-COVID-19 infection have complained of persisting symptoms, residual abnormalities and opacity in computed tomography (CT) scans, improper breathing, and continued loss of taste and smell [6]. Additionally, COVID-19 leads to impaired pulmonary function, specifically diffusion capacity and exercise capacity, which have been reported to last for months after recovery [3,7-9]. Moreover, many patients who recovered from COVID-19 were reported to have imaging abnormalities and even pulmonary fibrosis when discharged [2].

Patients who recovered from COVID-19 have shown a restrictive pattern of pulmonary function. Therefore, it is crucial to record the effects of COVID-19 after infection as it gives a better insight into the long-term consequences of COVID-19 [3]. The most severely impacted organ in COVID-19 is the lung, and the pathophysiological events include lung alveolar damage and destruction, which may result in vascular and alveolar remodeling and pulmonary hypertension or lung fibrosis. Therefore, assessment of lung injury and lung function damage in the early convalescence phase of patients with COVID-19 is essentially important [3]. Hence, in the present study, pulmonary functions were assessed in post-convalescent COVID-19 patients.

## Materials and methods

After approval from the Institutional Review Board (IRB) with reference number IRB/COM/STD/103/Oct-2021, a cross-sectional comparative study was performed among students and staff members of Gulf Medical University for a duration of one year from 2021 to 2022. The inclusion criteria consisted of both genders (58 were males and 42 were females) in the age range between 18 and 50 years, healthy adults, non-smokers who had never been infected by COVID-19, smokers for more than one year, those who had never been infected by COVID-19, non-smokers who tested negative for COVID-19 within one year post-recovery, and adults who had a history of smoking for more than one year and tested negative for COVID-19 within one year post-recovery. The exclusion criteria consisted of adults who had a previous history of any pulmonary infection, participants less than 18 years of age, history of chronic lung diseases, history of heart diseases, history of hemorrhagic or ischemic cerebrovascular diseases, history of chronic kidney diseases, adults from other universities, and participants not willing to participate. A non-probability convenient sampling method resulted in the inclusion of 100 participants for the current study based on the selection criteria. The study commenced after obtaining written informed consent from the participants who were categorized into four groups of 25 each. Group 1 involved 25 healthy adults who were non-smokers and who had never been infected by COVID-19; they were considered as the control group. Group 2 included 25 adults who had been smokers for more than one year and had never been infected by COVID-19. Group 3 consisted of a convalescent COVID-19 group of 25 adults, who were non-smokers and tested negative for COVID-19 within one year post-recovery. Group 4 involved 25 adults who were convalescent COVID-19 patients and were smokers who had a history of smoking for more than one year and tested negative for COVID-19 within one year post-recovery.

The questionnaire consisted of demographic information, height, weight, smoking habits, personal history, and duration after recovery from COVID-19. A spirometer (CONTEC SP70B handheld portable medical spirometer, China) and an oximeter (Pulse Oximeter, with LED SPO2, China) were the instruments used in the present study. Additionally, respiratory rate and pulse rate were monitored, and O2 levels were recorded using pulse oximeters. The participants underwent pulmonary function tests (PFTs), which were performed using a spirometer. The parameters included peak expiratory flow rate (PEFR) prediction, forced vital capacity (FVC) prediction, forced mid-expiratory flow rate (FEF_25-75%_), forced expiratory volume in the first second (FEV1) prediction, and FEV1/FVC ratio. The participants were instructed to sit on a chair, breathe through a mouthpiece that was closed tightly, and then exhale strongly after inhaling through the mouth as deeply as possible. The procedure was performed at least three times, and the best measurement was recorded and documented [10].

The data was recorded in Microsoft Excel (Microsoft Corp., Redmond, WA), and statistical analysis was performed using SPSS version 26 (IBM SPSS Statistics, Armonk, NY). Data analysis was performed using the one-way ANOVA test. The information was described as mean±standard deviation (SD). Using post hoc Tukey's honest significant difference (HSD), the differences in sample means were examined for significance, and a p-value of ≤0.05 was considered statistically significant.

## Results

A total of 100 participants were included in the current study consisting of staff members and students from Gulf Medical University. Gender distribution demonstrated that the majority of the participants were females with 58 in comparison to males with 42, and the mean age of the participants was 25±9 years. Additionally, the mean body mass index (BMI) of the participants was 25.02±4.62 kg/m^2^. Pulmonary function test parameters SpO2 levels, respiratory rate, and pulse rate of the participants divided into four groups are demonstrated in Table 1. In comparison to the convalescent COVID-19 group, the group consisting of convalescent COVID-19 smoking patients showed a significant increase (p=0.04) in FEV1/FVC%. However, no significant changes were observed between other groups (p>0.05). Moreover, the group consisting of convalescent COVID-19 smoking patients showed a significant increase (p=0.006 and p=0.045) in PEFR prediction when compared to the smoker group and the convalescent COVID-19 group, respectively.

**Table 1:** Study parameters The statistical test used was a one-way ANOVA test. Post hoc Tukey's HSD was utilized to examine the differences in sample means for significance, and the data was expressed as mean±SD. *p<0.05 versus the COVID-19 group, #p<0.05 versus the smoker group FEV1 = forced expiratory volume in one second, FVC = forced vital capacity, FEF_25-75%_ = forced mid-expiratory flow, PEFR = peak expiratory flow rate, SpO2 = oxygen saturation, ANOVA = analysis of variance, HSD = honest significant difference, SD = standard deviation

Parameters	Control group (n=25)	Smokers (n=25)	COVID-19 (n=25)	Smokers + COVID-19 (n=25)	p-value
FVC prediction	97.23±27.61	93.15±16.19	97.68±21.00	92.22±25.14	>0.05
FEV1 prediction	72.86±23.91	69.07±22.96	69.45±25.32	83.48 ±22.70	>0.05
FEV1/FVC%	67.11±21.60	64.90±23.54	61.97±25.01	79.63±21.96*	*0.04
FEF_25-75%_	66.14±43.51	57.92±33.62	57.63±29.07	82.19±32.71	>0.05
PEFR	50.96±30.06	41.20±23.19	46.40±26.32	66.60±27.38*#	*0.045, #0.006
SpO2	97.44±4.19	97.88±2.24	98.12±2.26	97.52±2.27	>0.05
Respiratory rate (breaths/minute)	17.84±2.93	18.16±2.86	16.44±2.81	18.20±3.08	>0.05
Pulse rate (beats/minute)	84.44±12.17	86.44±10.35	81.60±12.01	87.80±11.61	>0.05

**Figure 1:**
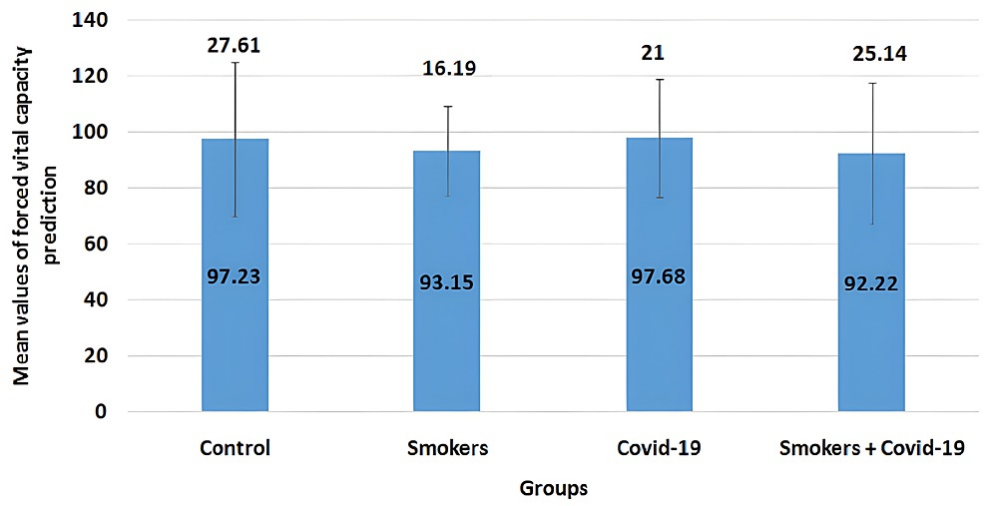
Mean values of forced vital capacity prediction The error bars are represented in terms of standard deviation. Smoker and convalescent COVID-19 smoking groups showed a non-significant decrease (p>0.05) when compared to convalescent COVID-19 and control groups.

The mean values of the FVC parameter in all four groups are illustrated in Figure 1. The mean values for the control group, smokers group, COVID-19 group, and smoker + convalescent COVID-19 group were 97.23±27.61, 93.15±16.19, 97.68±21.00, and 92.22±25.14, respectively. Smoker and convalescent COVID-19 smoking groups showed a non-significant decrease (p>0.05) when compared to convalescent COVID-19 and control groups.

**Figure 2:**
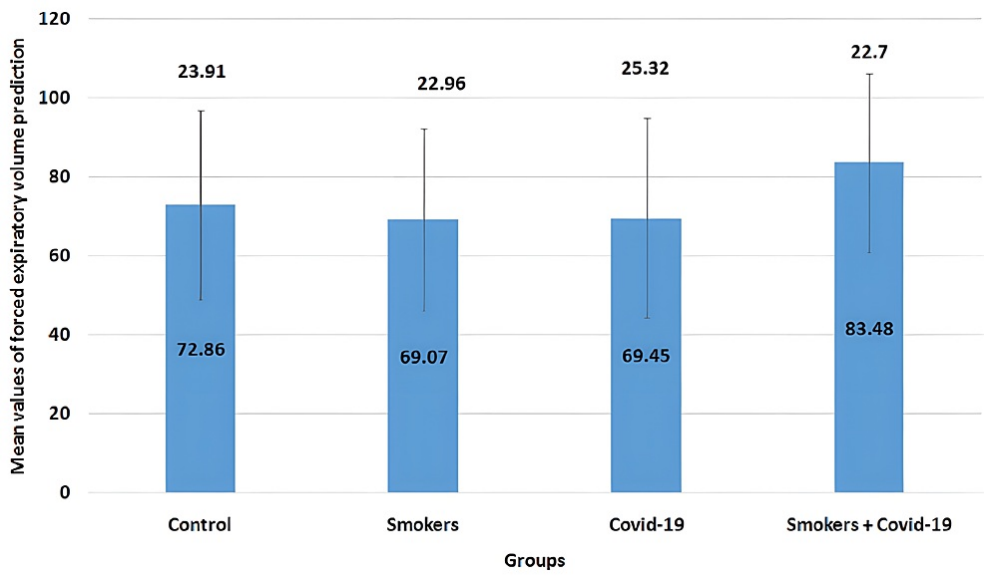
Mean values of forced expiratory volume in the first second prediction The error bars are represented in terms of standard deviation. Smoker and convalescent COVID-19 smoking groups showed a non-significant decrease (p>0.05) when compared to control groups. FEV1 = forced expiratory volume in one second

The mean values of the FEV1 parameter in all four groups are illustrated in Figure 2. The mean values for the control group, smokers group, COVID-19 group, and smoker + convalescent COVID-19 group were 72.86±23.91, 69.07±22.96, 69.45±25.32, and 83.48±22.70, respectively. Smoker and convalescent COVID-19 smoking groups showed a non-significant decrease (p>0.05) when compared to control groups.

**Figure 3:**
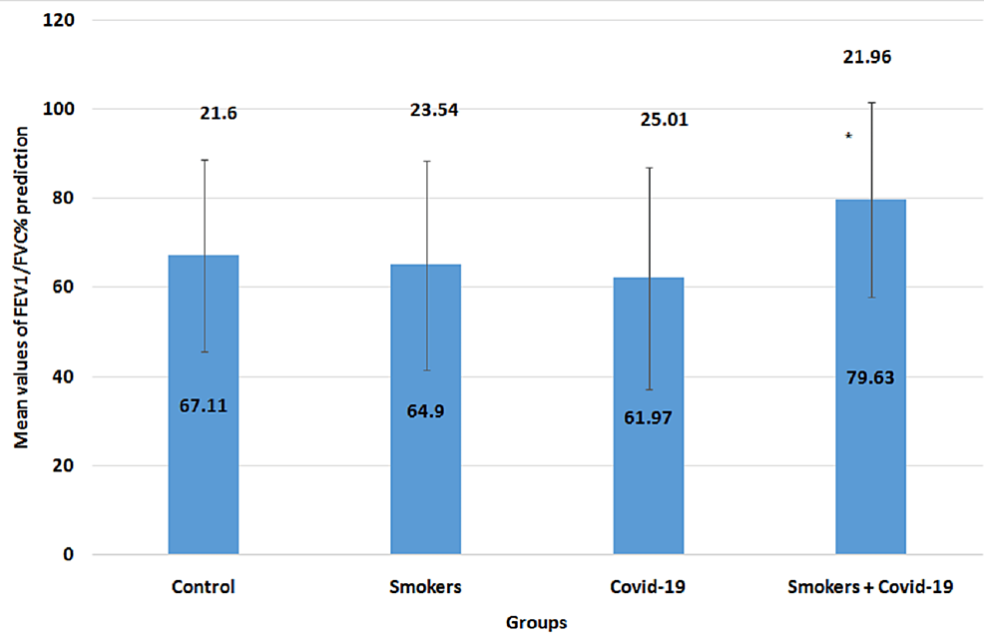
Mean values of FEV1/FVC% prediction The error bars are represented in terms of standard deviation. *p<0.05 versus the COVID-19 group: the convalescent COVID-19 smoking group showed a significant increase (p=0.04) when compared to the convalescent COVID-19 group. There were no significant changes between other groups (p>0.05). FEV1 = forced expiratory volume in one second, FVC = forced vital capacity

The mean values of the FEV1/FVC% parameter in all four groups are illustrated in Figure 3. The mean values for the control group, smokers group, COVID-19 group, and smoker + convalescent COVID-19 group were 67.11±21.6, 64.9±23.54, 61.97±25.01, and 79.63±21.96, respectively. The convalescent COVID-19 smoking group showed a significant increase (p=0.04) when compared to the convalescent COVID-19 group. There were no significant changes between other groups (p>0.05).

**Figure 4:**
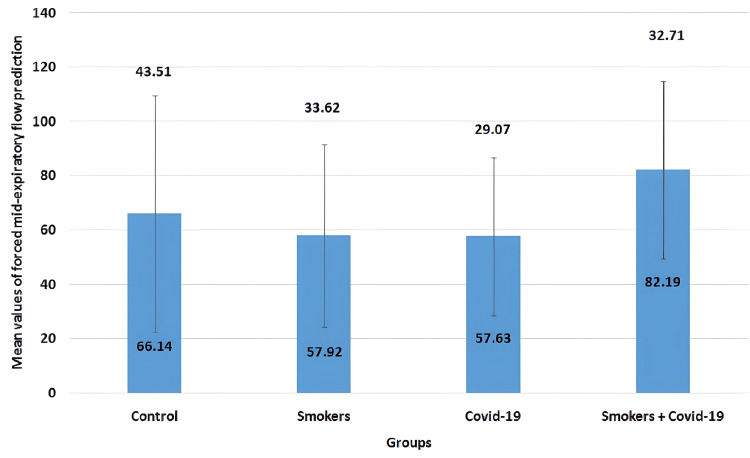
Mean values of forced mid-expiratory flow (FEF25-75%) prediction The error bars are represented in terms of standard deviation. There were no significant changes between the groups (p>0.05). FEF_25-75%_ = forced mid-expiratory flow

The mean values of the forced mid-expiratory flow (FEF_25-75%_) parameter in all four groups are illustrated in Figure 4. The mean values for the control group, smokers group, COVID-19 group, and smoker + convalescent COVID-19 group were 66.14±43.51, 57.92±33.62, 57.63±29.07, and 82.19±32.71, respectively. No significant changes were observed between the groups (p>0.05).

**Figure 5:**
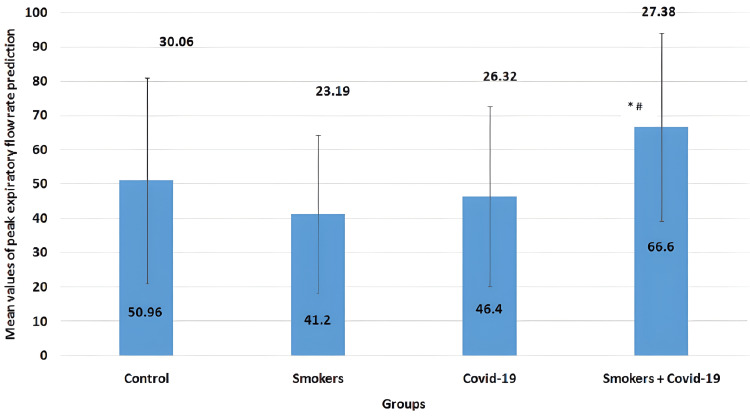
Mean values of peak expiratory flow rate The error bars are represented in terms of standard deviation. *p<0.05 versus the COVID-19 group and #=p<0.05 versus the smokers group: smokers and convalescent COVID-19 groups showed a non-significant decrease (p>0.05) when compared to the control group, but the convalescent COVID-19 smoking group showed a significant increase (p=0.006 and p=0.045) when compared to the smoker group and the convalescent COVID-19 group, respectively. PEFR = peak expiratory flow rate

The mean values of the PEFR parameter in all four groups are illustrated in Figure 5. The mean values for the control group, smokers group, COVID-19 group, and smoker + convalescent COVID-19 group were 50.96±30.06, 41.2±23.19, 46.4±26.32, and 66.6±27.38, respectively. Smokers and convalescent COVID-19 groups showed a non-significant decrease (p>0.05) when compared to the control group, but the convalescent COVID-19 smoking group showed a significant increase (p=0.006 and p=0.045) when compared to the smoker group and the convalescent COVID-19 group, respectively.

**Figure 6:**
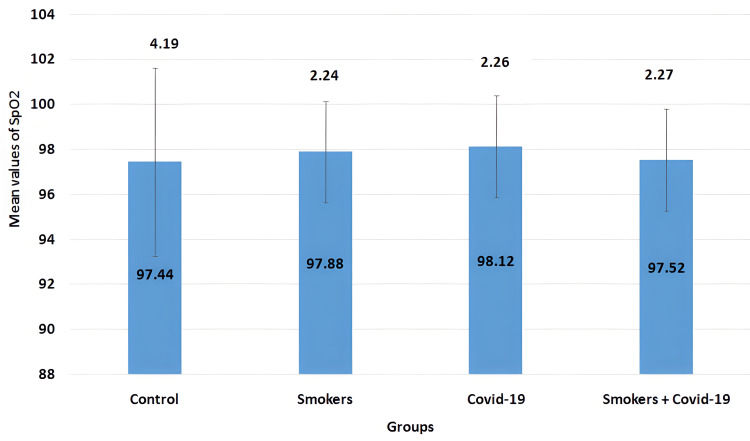
Mean values of SpO2 The error bars are represented in terms of standard deviation. There were no significant changes between the groups. SpO2 = oxygen saturation

The mean values of the SpO2 parameter in all four groups are illustrated in Figure 6. The mean values for the control group, smokers group, COVID-19 group, and smoker + convalescent COVID-19 group were 97.44±4.19, 97.88±2.24, 98.12±2.26, and 97.52±2.27, respectively. No significant changes were observed between the groups.

**Figure 7:**
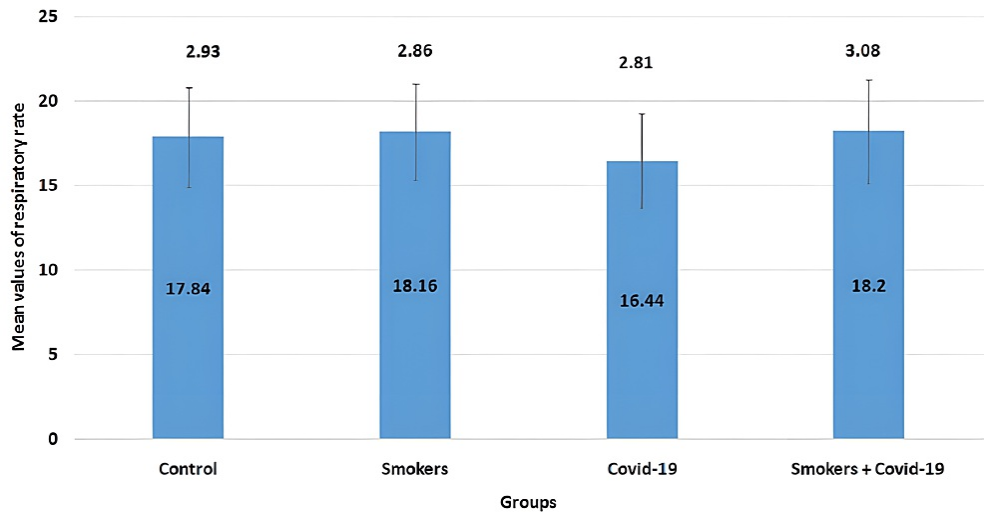
Mean values of respiratory rate The error bars are represented in terms of standard deviation. There were no significant changes between the groups.

The mean values of the respiratory rate parameter in all four groups are illustrated in Figure 7. The mean values for the control group, smokers group, COVID-19 group, and smoker + convalescent COVID-19 group were 17.84±2.93, 18.16±2.86, 16.44±2.81, and 18.2±3.08, respectively. No significant changes were observed between the groups.

**Figure 8:**
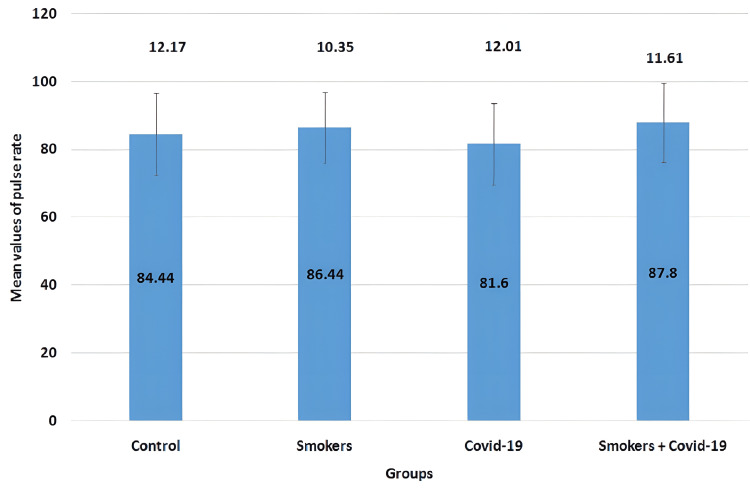
Mean values of pulse rate The error bars are represented in terms of standard deviation. There were no significant changes between the groups.

The mean values of the pulse rate parameter in all four groups are illustrated in Figure 8. The mean values for the control group, smokers group, COVID-19 group, and smoker + convalescent COVID-19 group were 84.44±12.17, 86.44±10.35, 81.6±12.01, and 87.8±11.61, respectively. No significant changes were observed between the groups.

## Discussion

The study highlighted the assessment of pulmonary functions in post-convalescent COVID-19 patients, smokers, post-convalescent COVID-19 smoking patients, and the control group. The study reported that most of the participants have healthy functioning of the pulmonary system, and the four groups showed no significant change in the parameters except for PEFR prediction values and FEV1/FVC ratio. Additionally, the findings described that there has been no significant change in FVC and FEV1 outcomes. Similarly, a previous study conducted by Lewis et al. [11] tested the pulmonary function of COVID-19 patients and concluded that there was no significant change in FVC and FEV1. This could be because most of the participants were not severely affected by COVID-19 and did not require hospitalization or due to the absence of chronic pulmonary disorders in the participants. Additionally, the present study was conducted on convalescent COVID-19 patients within one year post-recovery, which means that their lungs restored proper functioning with time. Another study conducted by Ye et al. [12] was found to be congruous with the present study in which it was observed that during infection, pulmonary function had been affected, but most of the participants showed gradual improvement during and post-recovery. Moreover, Liang et al. [13], in their three-month follow-up study involving survivors of coronavirus disease after discharge, showed that FVC and FEV were normal and only mild impairments were observed in some of the participants.

Additionally, in comparison to the control group, the results of the present study illustrated a non-significant (p>0.05) decrease in FEV1/FVC%, FEF_25-75%_ prediction, and PEFR prediction values in the smoker group and convalescent COVID-19 group. However, in comparison to the convalescent COVID-19 group, the group consisting of convalescent COVID-19 smoking patients showed a significant increase in FEV1/FVC ratio. Similar results were observed in the study conducted by Mogensen et al. [14], in which it was found that there was no evidence of mild to moderate COVID-19 affecting lung function in young adults and neither asthma nor allergic sensitization affected the results. Furthermore, the convalescent COVID-19 smoking group showed a significant increase in the FEV1/FVC ratio, which could be due to some restrictive pattern of the lung. Additionally, similar results were found in a study conducted by Fumagalli et al. [15], who reported that the FEV1/FVC ratio was increased in patients surviving COVID-19 pneumonia.

In peripheral airways where primary airflow blockage originates, the most sensitive measure of airflow is FEF_25-75%_. The non-significant change is most probably due to the non-affection of the tracheobronchial tree by COVID-19 infection. Additionally, no significant change was observed even in the smoker group as the sample involved a history of one year of smoking and above. The smoker's group showed no significant change in lung function test and respiratory rate when compared to the control group, which could be explained by the fact that the participants were recent smokers or due to the absence of other health issues that may interfere with the results. The study conducted by Dugral et al. [16] reported that smokers had better FVC and FEV1 values and significantly lower FEV1/FVC ratios in comparison to non-smokers. Another study conducted by Xavier et al. [17] showed that although smoking in general is harmful, the intensity of smoking plays a crucial role in determining the size of the damage done on the mucociliary clearance. Light smokers have less damage to the mucociliary clearance as compared to heavy smokers. Another study investigating the association of low-intensity smoking with respiratory risk conducted by Balte et al. [18] showed that the risk of death due to the presence of respiratory diseases among low-intensity smokers in comparison to non-smokers is almost not significant. The effect of low-intensity smoking is not as severe as heavy smoking, which could explain the fact that no correlation was found between pulmonary tests and smoking groups in the present study.

Furthermore, in the current study, pulse rate showed non-significant change among all four groups, which corresponded well with the study conducted by Linneberg et al. [19], which demonstrated the impact of smoking on resting heart rate and blood pressure and concluded that heavy smoking was associated with increased resting heart rate, which could explain the reason for why no change in pulse rate among the groups was observed in the present study since the participants involved were young and probably started smoking recently.

Strengths and limitations

As an objective metric for clinical assessment and monitoring of pulmonary outcomes of COVID-19 patients during recovery, lung function detection is a safe, non-invasive, and patient-friendly procedure. Patients' recovery may benefit from early pulmonary rehabilitation intervention, and pulmonary function may gradually improve over time. The limitations of the present study involved the recruitment of participants from a single university, a small sample size, and the inclusion of only young adults. Furthermore, none of the individuals had PFTs reported before COVID-19 infection. However, participants from various universities with large sample sizes and additionally involving older populations can be considered for the future scope of the study.

## Conclusions

Minor changes in lung function, primarily restrictive in nature, may be caused by COVID-19 in smoking patients and may partially last after recovery. The present study highlighted the assessment of PFTs among adults who were divided into four groups consisting of post-COVID-19 patients, smokers, post-COVID-19 smoking patients, and a control group. The study concluded that the status of PFTs was within the normal range in the post-convalescent COVID-19 and smokers groups, whereas convalescent COVID-19 smoking patients showed a significant increase in FEV1/FVC% and PEFR prediction values when compared to the convalescent COVID-19 group. This aids healthcare professionals in amending strategies to prevent consequences resulting from post-COVID-19 infection. Furthermore, future studies may be required with increased sample size in chronic smoker COVID-19 patients.
